# “Should I Further Engage in Staff Care?”: Employees’ Disclosure, Leaders’ Skills and Goal Conflict as Antecedents of Health-Oriented Leadership

**DOI:** 10.3390/ijerph20010162

**Published:** 2022-12-22

**Authors:** Sarah Pischel, Jörg Felfe, Laura Klebe

**Affiliations:** Department of Work, Organizational and Business Psychology, Helmut Schmidt University Hamburg, 22043 Hamburg, Germany

**Keywords:** health-oriented leadership, antecedents, disclosure, skills, goal conflict

## Abstract

Leaders play a crucial role in employees’ health and job satisfaction. When employees show early warning signs that their physical or mental health is at risk, leaders’ responsibility gains even more importance. Recent health-specific leadership approaches (health-oriented leadership; HoL) emphasize the importance of leaders ability to perceive employees’ warning signals (*staff care awareness*) to take appropriate action (*staff care behavior*). However, little is known about the factors facilitating or hindering the transfer from leaders’ awareness to concrete behaviors. In an experimental study (*N* = 91), we examined and manipulated antecedents of staff care behavior: (a) employees’ disclosure, (b) leaders’ HoL skills, and (c) leaders’ goal conflict in a 2 × 2 × 2 mixed factorial design. Employees’ disclosure and leaders’ skills were positively related to staff care behavior. Leaders’ goal conflict was not directly related to staff care behavior but had an indirect effect and diminished the positive relationship between disclosure and staff care behavior. The findings deepen the theoretical understanding of the HoL concept. By studying the influence of employees’ disclosure on staff care behavior, our study complements a follower-centered perspective. We provide practical recommendations for workplace health promotion and how leaders’ staff care behavior can be fostered.

## 1. Introduction

Leaders play a crucial role in employees’ health and work-related outcomes such as work performance, engagement, or satisfaction [1,2]. When employees show early warning signs that their physical or mental health is at risk (e.g., mood swings, social withdrawal, a decline in performance), leaders’ responsibility gains even more importance [3]. Leaders can provide direct or indirect support at an early stage (e.g., through conversations, workplace adjustments, or improved self-care; [2,4,5]) and thus prevent worsening or chronification [6,7].

Recent health-specific leadership approaches such as health-oriented leadership (HoL; [8]), therefore, emphasize the importance of leaders’ ability to perceive employees’ warning signals (staff care *awareness*) in order to take appropriate action (staff care *behavior*, e.g., reducing workload, adjusting tasks to reduce demands, and encouraging to participate in workplace health promotion). Although awareness and behavior are related [8,9], it cannot be taken for granted that the perception of warning signals will automatically lead to appropriate action. For example, although leaders may have noticed behavioral changes, there may be difficulties that hinder further staff care behaviors. We are not aware of any study investigating the factors that may influence the transition from staff care awareness to concrete staff care behavior. Instead, so far, empirical research has focused on antecedents of overall staff care independent of employees’ health status (e.g., [10]). From a theoretical and practical perspective, a deeper understanding of which factors facilitate or hinder the transfer from leaders’ awareness to concrete behaviors is needed.

When leaders notice warning signals, they may clarify the meaning of their perception in a personal discussion with the respective employee before taking further actions, as warning signals may sometimes be less clear or ambiguous and be both a sign of an emerging mental health issue or just poor motivation [11,12]. However, several difficulties may exist in this conversation that may ultimately hinder further staff care behavior. Drawing upon the ability, motivation, opportunity (AMO) framework for predicting work performance and transferring it to predicting staff care behavior as an important part of leadership performance, we propose three factors: leaders’ skills, leaders’ motivation, and the opportunity and permission from the employee in terms of disclosure [13,14].

The first challenge leaders may face in the conversation about noticed warning signals is that employees are not willing to disclose their problems. As mental problems and illnesses are still stigmatized [15,16,17], many employees decide not to talk about their mental health problems at work, even when asked [18]. If an employee conceals an existing mental problem (e.g., denial, trivialization), there may be a risk that the leader will misinterpret the employee’s warning signals. For example, leaders may misinterpret behavioral changes (e.g., reduced performance) as laziness or lack of motivation, so that leaders increase pressure instead of offering support [19]. Until now, it remains unclear whether employees’ non-disclosure hinders further staff care behavior.

The second challenge for leaders may be uncertainty about how to address the issue appropriately. Studies show that many leaders feel uncomfortable or ‘out of their depth’ in conversations with employees about mental health due to a lack of knowledge and skills [20,21,22]. When leaders’ have no confidence and prior health-oriented leadership skills (e.g., knowledge of typical warning signals, stigma, disclosure, opportunities to support when employees’ health is at risk), they may be more likely to avoid discussing health-related topics in detail and thus may also not show additional supportive behavior. In contrast, when leaders are aware that they are also responsible for employees’ health, have an understanding of mental health issues, and have confidence in what behavior is appropriate, they are more likely to display more staff care behavior. Moreover, knowing that concealing is one typical reaction of employees in a conversation about noticed warning signals, leaders can better classify this reaction and decide on staff care behavior independent of the employees’ reaction. That is, the negative effect of non-disclosure on staff care behavior may decrease when health-oriented leadership skills are higher. It is important to know whether providing critical knowledge and skills may already have a positive impact and may also influence the effect of non-disclosure on staff care behavior.

The third challenge for leaders may come into play when an affected employee is particularly important for the achievement of current goals. If an employee develops a mental health problem and is highly important for the current goal achievement (e.g., a long-standing project manager in a software company), the leader may have to decide whether he or she puts the health of the respective employees ahead of the organizational goals. More precisely, experiencing a goal conflict may minimize the leader’s motivation for health-promoting behavior toward the high performer [20,23]. Even when the indispensable employee tells the leader that he or she is struggling with mental health, the leader may be more inclined to refrain from staff care behavior. That is, the positive relationship between disclosure and staff care behavior may be weakened by the leader’s reduced motivation due to the goal conflict. To date, it is unclear whether leaders’ experience of a goal conflict affects their staff care behavior and also influences the relationship between employees’ disclosure and staff care behavior.

**Figure 1:**
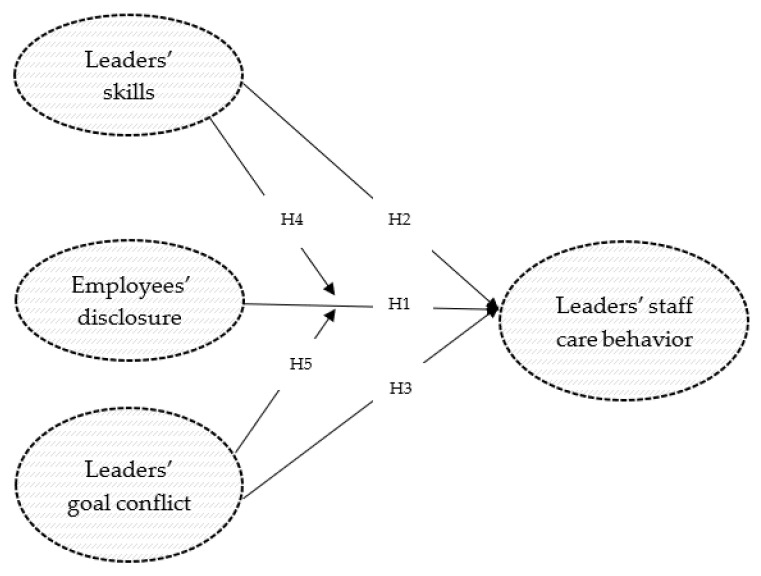
Research model.

With this study, we aim to fill previous gaps and deepen the understanding of antecedents of health-oriented leadership behavior given the situation where a leader perceives clear warning signals and starts a conversation about his or her perception of behavioral changes with the respective employee. In an experimental study, we manipulated (a) employee disclosure (*concealing* vs. *revealing mental health problems*), and (b) leaders’ skills (*short psychoeducational video (intervention group)* vs. *video about functions in Microsoft Teams (control group)*) to examine their direct effects and interaction on staff care behavior. In addition, we manipulated c) the experience of a goal conflict (*low* vs. *high employee importance for the leader’s current goal achievement*) to investigate the direct effect of goal conflict on subsequent staff care behavior and the indirect effect of goal conflict on the relationship between disclosure and staff care behavior (see the research model in Figure 1).

This study makes several theoretical and practical contributions: (a) adding to the health-specific leadership literature [8], (b) applying the AMO framework to studying antecedents of leadership behavior, (c) complementing a follower-centered perspective by focusing on the active influence of employees on leadership behavior [24], (d) deriving recommendations for organizations that aim at fostering health-oriented leadership given that employees health may be at risk.

## 2. Theoretical Background

### 2.1. The Concept of Health-Oriented Leadership

As a complement to a growing body of research on the link between broader, more general leadership constructs, such as transformational leadership and employees’ health (e.g., [25]), the HoL concept was developed to capture health-specific leadership attitudes and behaviors [8]. Research has shown that health-oriented leadership relates to employees’ health above and beyond transformational leadership and leader-member exchange [8,26,27].

The HoL concept emphasizes the joint responsibility of leaders and followers for health. *Self care* refers to leaders’ and employees’ concern for their own health [8]. *Staff care* refers to leaders’ concern for their employees’ health. Self care and staff care both consist of three subfacets, namely (1) *value* (2) *awareness,* and (3) *behavior*. *Value* comprises the importance leaders attach to employee health. *Awareness* defines leaders’ ability to perceive employees’ warning signals of emerging (mental) health issues. *Behavior* encompasses fostering healthy working conditions and minimizing health risks (e.g., taking regular breaks, enhancing work organization, and supporting participation in occupational health programs).

Staff care is directly related to various employee outcomes, including better general health as well as lower strain, physical health complaints, burnout, depression, and anxiety (e.g., [28,29,30,31]). Staff care also indirectly influences employees’ well-being by fostering employees’ self care and by enhancing the team climate and working conditions [32,33,34].

Recently, researchers have shifted their focus from outcomes to antecedents of staff care. An understanding of antecedents is not only essential to gain insights into how to foster health-oriented leadership in practice but also for the current research debate that aims at advancing the theory of healthy leadership [35]. Thus far, empirical evidence demonstrated that leader variables (e.g., strain, self care; [10,12,36]), context variables (e.g., autonomy, organizational health climate, high-performance work practices; crisis; [9,10,36]) as well as follower variables (e.g., employees’ strain, clarity of warning signals; [12,36]) play a crucial role in staff care.

Although leaders become even more important when employees’ health is at risk [34], it cannot be taken for granted that the perception of warning signals will automatically lead to appropriate action. Little is known about the antecedents of health-oriented leadership behavior given the situation where an employee has displayed warning signals.

### 2.2. Antecedents of Staff Care Behavior

#### 2.2.1. Ability, Motivation, Opportunity (AMO) Framework

The AMO framework postulates that high employee performance is a result of employees’ abilities (knowledge, skills), motivation, and opportunities to use their abilities and motivation [13,14,37]. The model itself was developed to predict employee work performance [13] and was later applied to the field of human resource management (HRM). The practical recommendation for HRM aiming at maximizing employee performance was to enhance employee skills, motivation, and opportunity to contribute (e.g., extensive training, rewards, information sharing; [38]). In this study, we apply and adapt the AMO framework to leadership behavior as an important part of their performance. Following the three predictors in the AMO framework [13,14,37], we focus on three antecedents of staff care behavior that may be especially relevant in the situation where a leader perceives that an employee displays warning signals (staff care awareness) and wants to take further health-oriented actions (staff care behavior) in a next step. These three antecedents are (1) leaders’ health-oriented leadership skills, (2) leaders’ (reduced) motivation due to the experience of a goal conflict (employee health vs. the importance of the employee for the leader’s goal achievement), (3) employees’ disclosure that serves as the opportunity and permission for leaders to use their skills and motivation.

#### 2.2.2. “I Feel Emotionally Drained and I Could Need Help”—Effects of Employees‘ Disclosure on Staff Care Behavior

Employees decide to conceal or reveal their problems after a cost-benefit analysis of anticipated consequences [39]. According to the well-known disclosure model by Ragins [39], those anticipated consequences are stigma, internal psychological factors (e.g., centrality to self-concept), and environmental factors (e.g., supportive relationships). Common stigmas associated with mental illness include labels such as being incompetent, dangerous, unpredictable, unstable, and weak [5,16]. As a consequence of anticipating stigma, many employees decide to conceal an existing mental problem [39].

However, although concealing may protect from labeling, social exclusion, or other discriminations, empirical evidence demonstrates several negative consequences (e.g., [18,39,40]). In addition to an increase in stress and intrusive thoughts, which may diminish work performance or satisfaction [39,40], concealing inhibits further helping behaviors of others (e.g., instrumental or psychosocial support [40]). Up to now, it is open whether concealing also affects concrete health-specific leadership behaviors. Although previous research has focused on leadership as an important precondition for disclosing mental problems (e.g., [27]), the reverse causality has so far been neglected.

Drawing upon disclosure models, previous empirical evidence on the positive relationship between helping behavior and employees’ disclosure [39,40], and in line with the follower-centered perspective [24], we expect a direct positive effect of employees’ disclosure on further staff care behavior. Becoming aware of warning signals, leaders may first share their perception with the respective employee in a conversation and clarify the cause for the ambiguous noticed signals (e.g., health-related problems or non-health-related problems). As described by Martin [41], “[…] handling initial discussions about the employee’s condition set[s] the tone for subsequent interactions” (p. 57). When an employee decides to hide an existing mental problem due to anticipated stigma and instead trivializes the mentioned behavioral changes (e.g., excuses such as bad weather, problems with children), leaders may believe the employee or even search for alternative explanations (e.g., loss of motivation). As a result, leaders may even increase pressure instead of offering further health-oriented support [19]. In contrast, when employees reveal their mental problems, leaders can correctly classify the noticed changes as signs of an employee’s emerging mental health issue and then offer tailored support. Following the adapted AMO framework, employees’ disclosure may also serve as a permission and an opportunity that encourages further staff care behavior as mental health issues are considered a highly sensitive topic [13,23].

**H1.**  *Employees’ disclosure is positively related to subsequent staff care behavior: While employees‘ disclosure fosters staff care behavior, concealing reduces staff-care behavior.*

#### 2.2.3. “I Know How I Can Handle This Situation”—Effects of Health-Oriented Leadership Skills on Staff Care Behavior

In addition to employees’ disclosure, further staff care behavior may also be contingent on leaders’ health-oriented leadership skills and knowledge. In practice, most leaders feel they are lacking health-oriented leadership skills and knowledge to successfully handle situations with employees with emerging mental health issues [20,21,22].

However, following the adapted AMO framework, leaders’ abilities, knowledge, and skills are essential antecedents of their behavior [13]. Specific knowledge to manage employees with emerging mental health issues encompasses the responsibility of leaders for employees’ health, as well as an understanding of mental health issues, typical warning signals, stigma, and typical reactions of employees in conversations about noticed warning signals (i.e., concealing vs. revealing). Finally, it also includes appropriate leadership behavior [23,41]. Leaders are not naturally born with that knowledge and skills, but their knowledge can be developed and improved through education and training [37,42]. Although leaders’ skills are considered an important antecedent of further behavior, it yet remains unclear whether health-oriented leadership skills provided by short interventions may already have a positive impact.

In line with the AMO framework, we predict that leaders with health-oriented leadership knowledge may not only feel more confident talking to their employees about health issues but also have an idea of what health-oriented leader behavior they can provide in the next step. In contrast, when leaders have no prior health-oriented leadership skills, they may feel ‘out of their depth’ and thus avoid discussing health-related topics in detail. As they are insecure about what behavior is appropriate, they may also refrain from engaging in additional health-oriented behavior. Due to missing knowledge, leaders may even regard employees’ health as a private matter and abdicate from their responsibility as a leader [23].

**H2.**  *Leaders’ health-oriented leadership skills are positively related to subsequent staff care behavior.*

#### 2.2.4. “I Can’t Successfully Reach My Goals without You! I Need You Now”—Effects of Leaders’ Experience of a Goal Conflict on Staff Care Behavior

Achieving goals is a core function of leadership. Leaders are highly motivated to achieve organizational goals, but at the same time, there is a motivation to maintain and improve followers’ health [23,43]. In some instances, striking a balance between the organizational needs and the needs of employees may be easily established as needs coincide (e.g., the company’s goal for sales growth and the employee’s goal to obtain profit-sharing). In other instances, there may exist conflicting needs and goals. When an employee with high importance for the leader’s current goal achievement (e.g., a sales manager in an organization with high sales growth goals) develops a mental health issue and displays warning signals such as diminishing performance or social withdrawal, it may be hard for leaders to decide how to balance the different needs. If the health of an important employee is at risk, leaders may want to adjust tasks, minimize workload or grant more break times to reduce demands. However, this can jeopardize the achievement of the organizational goals.

When leaders experience a goal conflict, the willingness to display staff care may decrease. Goal conflicts are associated with several negative outcomes, including lower motivation, greater levels of negative affect, and increases in depression, anxiety, and stress [44]. In turn, leaders’ stress has been linked to an increase in destructive and a decrease in constructive leadership [36,45,46]. That is, due to the goal conflict, leaders’ motivation to display staff care may be diminished. Instead, they may want to withdraw and avoid further employee interactions or even react with anger and pressure instead of showing additional health-promoting behavior (cross-over effect; [47]). Short-term goal achievement is prioritized over longer-term health consequences. Following this line of reasoning, leaders would be more motivated to display staff care behavior to an employee who is less important for current goal achievement because performance losses are less crucial and may be compensated more easily. A lower goal conflict and thus no additional stress is expected. Leaders can replace the less important employee more easily so that organizational goals are still achieved.

Therefore, we hypothesize that leaders are less motivated to show staff care behavior if they experience a goal conflict. The goal conflict (employee health vs. the indispensability of the employee for the leader’s goal achievement) diminishes leaders’ motivation while stress increases. Following the AMO framework, reduced motivation leads to less staff care behavior [20,23].

**H3.**  *The experience of a goal conflict (employee health* vs. *the importance of the employee for the leader’s goal achievement) is negatively related to subsequent staff care behavior.*

In addition to direct effects, there may also be interaction effects between disclosure, skills, and goal conflict. As stated in H1, employees‘ disclosure may foster the following staff care behavior, and concealing reduces further staff-care behavior. However, the negative relationship between concealing and staff care behavior may be weakened by leaders’ health-oriented leadership skills. That is, the knowledge of typical employee reactions (concealing as a frequent reaction of employees due to stigma) may leave leaders with more confidence to display staff care behavior independent of the employees’ reaction.

**H4.**  *The negative relationship between concealing and subsequent staff care behavior is moderated by leaders’ health-oriented leadership knowledge and skills. The relationship is weaker for higher knowledge and skills.*

In addition, the positive relationship between disclosure and staff care behavior (H1) may be weakened when leaders experience a goal conflict. That is, when an indispensable employee discloses an existing mental health problem and the leader experiences a goal conflict as organizational goals still need to be achieved, leaders may overhear this important health information, ignore it, or trivialize its significance. As a consequence, the leader may be more inclined to refrain from further staff care behavior.

**H5.**  *The positive relationship between disclosure and subsequent staff care behavior is moderated by leaders’ experience of a goal conflict. The relationship is weaker when leaders experience a goal conflict.*

## 3. Method

### 3.1. Design

To examine the influence of disclosure (H1), health-oriented leadership skills (H2), goal conflict (H3), the interaction between disclosure and skills (H4), and the interaction between disclosure and goal conflict (H5) on leaders’ staff care behavior, an experimental study was conducted (see the research model in Figure 1). We systematically manipulated employees’ disclosure (within factor 1), goal conflict (within factor 2), and health-oriented leadership skills (between factor 3) as independent variables, resulting in a 2 (*concealing* vs. *revealing*) × 2 (*no goal conflict* vs. *goal conflict*) × 2 (*intervention* vs. *control group*) mixed factorial design. Combining the two within factors resulted in four vignettes that were presented to a group with intervention and a control group (between factor health-oriented leadership skills). The dependent variable was staff care behavior.

### 3.2. Sample

We conducted the experiment as an online vignette study from May to August 2022 and recruited *N* = 91 participants with a mean age of 26.70 years (*SD* = 8.94, range 19–60) via convenience sampling (see Table 1 for sample characteristics). Most of the participants were male (56%) and had a German higher education entrance qualification ((52.7%), university degree (40.7%), secondary school (4.4%), vocational training (1.1%), polytechnic degree (1.1%)). The sample had a mean working experience of *M* = 6.90 years (*SD* = 8.93), and 28.6% of participants reported having leadership experience (*M* = 3.04 years; *SD* = 4.23).

### 3.3. Procedure and Materials

After giving their informed consent, participants were asked to take the role of a leader in an automotive company being responsible for a project team with 15 employees. Against the background of the market launch of an electric car, the project team takes care of the financing and distribution of the car (e.g., attracting investors, securing financing, marketing, communication with car dealers). The leader notices that four members of the team are “struggling” and displaying behavioral changes. Therefore, the leader decides to conduct conversations with each of the four employees.

First, before the conversations with the four employees, participants had to check emails as a basis of the leader’s daily routine. The latest email was from the human resource department (HR) with a short video. As a cover story, participants were told that HR regularly sends out short videos (e.g., about leadership, working from home, software) as leaders are required to earn educational points. Participants were told that there was still some time before the employee conversations, so they were given the task of watching the video and collecting educational points by answering some post-video questions correctly. Participants randomly either watched a psychoeducational video (intervention group) or a video about functions in Microsoft Teams (control group) to manipulate health-oriented leadership skills (factor 3). Following Martin et al. [41] and Vonderlin et al. [42], the psychoeducational video provided knowledge about health-oriented leadership (staff care components), typical warning signals, stigma, disclosure, and ways to further address and support employees with health risks (embedded in a case study).

Second, in preparation for the employee conversations, participants were given the task of checking the monthly report that contained all annual project goals with their current level of goal achievement. The report indicated that two goals have not yet been satisfactorily met ((1) acquisition of investors; (2) number of orders required and order volume) but must be achieved soon to ensure the announced product launch. We provided participants with the report so that they could experience a goal conflict in the employee conversations depending on the importance of the respective employee for goal achievement.

Third, participants started reading the four vignettes. Each vignette represented a conversation with one of the four struggling employees. In the vignettes, participants were first provided with information about the employee’s personal strengths and past successful work results. The next section contained information about the employee’s position, tasks, and current behavioral changes (warning signals). All four employees displayed warning signals of an emerging depression (e.g., exhaustion, social withdrawal, reduced performance) derived from Martin et al. [23]. Employees’ importance for the leader’s current goal achievement was either low (“*Mr. Mueller is mainly responsible for assistance tasks.*”) or high (“*Mr. Messner is the expert for marketing and sales in your team. In this phase of the project, his expertise is essential and very important for you to achieve the required order volume.*”). A goal conflict (factor 2) should occur for employees with high importance as they are indispensable and cannot simply be replaced. The next section contained an extract from the beginning of the conversation with the respective employee, in which disclosure (factor 1) of the employee was either concealing (“*Although you made every effort to provide Mr. Mueller with a trusting environment, he avoided the questions about the noticed behavioral changes. Mr. Mueller said that he was currently being troubled by the changes in the weather, but that everything else was fine.*”) or revealing (“*Mr. Meister opened up to you that he was not doing very well at the moment. He feels like a failure and is ashamed because he keeps making mistakes and thus spends an unnecessary amount of time on his tasks and thus also causes colleagues extra work. (…) In the meantime, he has reached a point where he does not know what to do on his own. Therefore, he asks you for help*.”). After reading each vignette, participants had to rate their following staff care behavior. All vignettes were similar in length and presented in a randomized order to avoid sequence effects.

Materials were pretested to check their validity. Participants (*N* = 24) rated on a five-point scale (1) the level of employees’ disclosure (1 = *not willing to open up at all* to 5 = *very willing to open up*), and (2) the perception of a goal conflict (1 = *not indispensable for current goal achievement at all* to 5 = *highly indispensable for current goal achievement*). All ratings were as expected, indicating that manipulation was successful and that participants perceived higher disclosure for disclosing employees (*M* = 4.75, *SD* = 0.36) than for non-disclosing employees (*M* = 1.54, *SD* = 0.49); *t*(23) = 29.66, *p* < 0.001). Moreover, participants perceived a goal conflict and higher indispensability for employees with project tasks that contribute to the yet unfulfilled goals (*M* = 4.79, *SD* = 0.33) than for employees with project tasks that do not contribute to the yet unfulfilled goals (*M* = 3.21, *SD* = 0.59; *t*(23) = 12.52, *p* < 0.001).

### 3.4. Measures

#### 3.4.1. Staff Care Behavior

We used seven items from the health-oriented Leadership instrument [48] to measure staff care behavior. A sample item was “*You try to reduce your employee’s demands by optimizing work routines (e.g., redistribution of tasks, support of daily planning)*”, rated on a five-point scale ranging from 1 (*not at all true*) to 5 (*completely true*). Cronbach’s α ranged between 0.70 and 0.81.

#### 3.4.2. Attention Check

In addition to telling participants to read the vignettes conscientiously, we included four attention checks to test whether participants read the scenarios carefully throughout the experiment. Sample questions were: “*In which professional area does Mr. X work?*”. We defined an adequate level of attention by answering three of four checks correctly.

### 3.5. Statistical Analyses

H1 to H5 were tested with a three-way mixed repeated measure ANOVA with the two within-subject factors disclosure (*concealing* vs. *revealing*), goal conflict (*no goal conflict* vs. *goal conflict*), and the between-factor health-oriented leadership skills (*intervention* vs. *control group*). We referred to η_p_^2^ as an indicator of the effect size. Values of 0.010 indicate a small effect size, while 0.059 represents a medium, and 0.138, a large effect size [49].

## 4. Results

### 4.1. Preliminary Analyses

No participants had to be excluded due to attention checks, as no participant gave more than one incorrect answer. A MANOVA showed that men and women did not differ with respect to their staff care behavior *F*(8, 86) = 1.16, *p* = 0.335, η_p_^2^ = 0.051, Wilk’s Λ = 0.949 and that age was not significantly related to staff care behavior (*r* = −0.07 to −0.12, *p* = 0.259 to 0.485). Therefore, we did not include gender and age in our analyses. The means and standard errors of the focal variables for each condition are listed in Table 2.

### 4.2. Effect of Employees’ Disclosure on Staff Care Behavior

H1 postulated that staff care behavior is higher when employees reveal their mental health condition. There was a significant main effect for disclosure, *F*(1, 89) = 91.15, *p* < 0.001, η_p_^2^ = 0.501, indicating a large effect size. In line with expectations, Bonferroni-adjusted post-hoc analysis revealed that participants were more likely to display staff care behavior for revealing employees (*M* = 4.40, *SE* = 0.05) than for concealing employees (*M* = 4.02, *SE* = 0.06; ∆ = 0.38, 95% CI [0.30; 0.46], *p* < 0.001). Therefore, H1 is supported.

### 4.3. Effect of Health-Oriented Leadership Skills on Staff Care Behavior

H2 postulated that staff care behavior is higher when leaders have higher health-oriented leadership skills (intervention group). There was a significant main effect for group, *F*(1, 89) = 4.01, *p* < 0.05, η_p_^2^ = 0.043, indicating a small effect size. In line with expectations, Bonferroni-adjusted post-hoc analysis revealed that participants showed higher staff care behavior in the intervention group (*M* = 4.32, *SE* = 0.07) than in the control group (*M* = 4.11, *SE* = 0.08; ∆ = 0.21, 95% CI [0.02; 0.42], *p* < 0.05). Therefore, H2 is supported.

### 4.4. Effect of Leaders’ Experience of a Goal Conflict on Staff Care Behavior

H3 postulated that staff care behavior is higher when leaders experience no goal conflict. However, against the assumptions, there was no significant main effect of goal conflict on staff care behavior, *F*(1, 89) = 1.08, *p* = 0.302, η_p_^2^ = 0.012. Therefore, H3 is rejected.

### 4.5. Moderating Effect of Health-Oriented Leadership Skills on Relationship between Employees’ Disclosure and Staff Care Behavior

H4 expected that the association between disclosure and staff care behavior is weaker for higher skills. Contrary to our expectations, there was no significant interaction between skills and disclosure, *F*(1, 89) = 0.72, *p* = 0.398, η_p_^2^ = 0.008. Therefore, H4 is rejected.

### 4.6. Moderating Effect of Leaders’ Experience of a Goal Conflict on Relationship between Employees’ Disclosure and Staff Care Behavior

**Figure 2:**
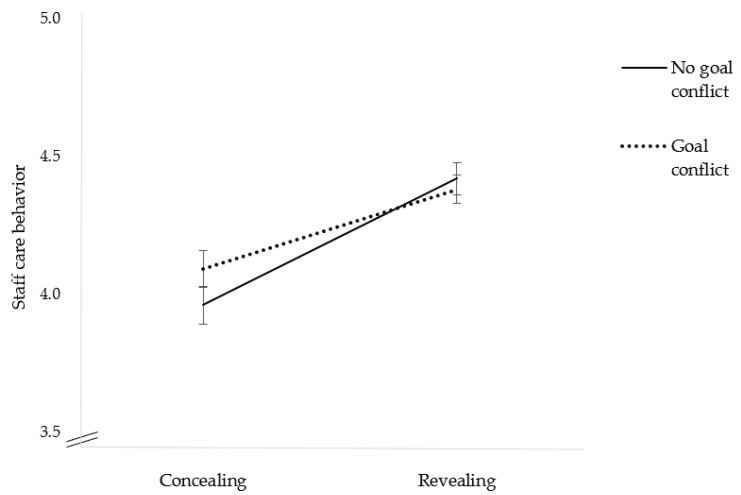
Interaction Effect Between Disclosure (Concealing vs. Revealing) and Goal Conflict (No Goal Conflict vs. Goal Conflict) on Staff Care Behavior.

H5 expected that the association between disclosure and staff care behavior is weaker for the experience of a goal conflict. In line with the assumption, there was a significant interaction between goal conflict and disclosure, *F*(1, 89) = 6.90, *p* < 0.05, η_p_^2^ = 0.072, indicating a medium effect size. The relationship was weaker when leaders experienced a goal conflict (see Figure 2). Therefore, H5 is supported. Table 3 shows the results of the three-way mixed repeated measure ANOVA.

## 5. Discussion

Leaders play an essential role in employees’ health and work-related outcomes such as performance, engagement, and satisfaction. When leaders recognize through warning signals that their employees’ health may be at risk (*staff care awareness*), leaders can take concrete health-promoting actions (*staff care behavior*) to prevent worsening or chronification. However, awareness is not always translated into further actions. Until now, little is known about the factors facilitating or hindering the transfer from staff care awareness to concrete staff care behaviors. The purpose of this study was to expand our understanding of staff care behavior by examining employees’ disclosure, leaders’ skills, and leaders’ goal conflict as antecedents following the adapted AMO framework [13,14].

First, we expected and provided evidence that employees’ disclosure fosters further staff care behavior. Our findings support prior studies showing a positive relationship between helping behavior and employees’ disclosure [39,40]. However, our study extends previous research by examining concrete health-specific leadership behaviors. In addition, by demonstrating the causal effects of disclosure on staff care behavior, we add to previous calls of conducting more experimental studies in leadership research [50]. Integrating our findings with a previous study that showed that staff care facilitates disclosure [27], we may conclude that there exists a bidirectional relationship between disclosure and staff care: disclosure enables staff care, but staff care also facilitates disclosure. By addressing followers’ behavior as an antecedent of leaders’ behavior, our study complements a follower-centered perspective [24]. That is, we may conclude from our findings that employees have a direct influence on leadership behavior when leaders depend on employee information. However, this influence may bring a “dark side” and serious negative consequences for employees fearing to open up because of stigma [15,16,17]. Although concealing employees may need the same support as revealing employees, leaders may show less staff care behavior toward the first group. In line with our adaption of the AMO framework to study antecedents of staff care behavior, leaders with concealing employees may lack an opportunity or permission that encourages further staff care behavior [13,23]. Another possible explanation is that leaders may rather misinterpret noticed behavioral changes (e.g., as a lack of motivation; [19]), resulting in an increase in pressure instead of support.

Second, drawing upon the AMO framework, we hypothesized and found that health-oriented leadership skills further enhance staff care behavior. This finding is in line with empirical evidence on the positive effects of education and training on further staff care behavior [37,42].

Third, drawing upon the AMO framework and empirical evidence on the negative effects of goal conflicts, we expected that the experience of a goal conflict reduces leaders’ motivation, which then diminishes further staff care behavior. Contrary to our expectations, we found no support that leaders’ experience of a goal conflict is directly related to their staff care behavior. Although previous studies found that goal conflicts are associated with lower motivation and increased stress [44], which in turn have been associated with a decrease in constructive leadership [45,46], we found that leaders showed staff care behavior independent of the employee’s importance for current goal achievement. Our findings reveal that leaders do not risk employees’ health due to the goal pressure they experience. Instead, when leaders perceive employees’ warning signals, they have a high motivation for enhancing employees’ health and thus show the same amount of staff care behavior to employees. This finding is also in line with a recent study by Klebe and colleagues [36], showing that employee strain is positively related to leaders’ staff care. The authors explain this finding with COR theory [51]: leaders engage in more staff care when employees are strained as they want to protect employees’ health (gain more resources and prevent losses in the long term). We expand this finding by demonstrating that leaders do not make a difference between strained employees with high or low importance for current goal achievement. That is, the experience of a goal conflict has no detrimental effects on the positive relationship between employee strain (perception of warning signals) and staff care behavior.

However, we found that a goal conflict has an indirect effect on the relationship between disclosure and staff care behavior. In line with our expectations, we found that the experience of a goal conflict mitigates the relationship between disclosure and staff care behavior. More precisely, we expected that the effect is stronger and weakened on the side of high disclosure: When an indispensable employee tells the leader that he or she is struggling with mental health, the leader may be more inclined to refrain from staff care behavior. Somewhat contrary to our expectations, we found that when disclosure was high, participants showed similar staff care behavior independent of the experience of a goal conflict. When disclosure was low, participants showed higher staff care when experiencing a goal conflict. To the best of our knowledge, this is the first study that investigated the interplay between goal conflict and disclosure on staff care behavior. We may conclude that leaders are more sensitive to important employees, even when they do not open up. At the same time, there is a risk that concealing employees who are less important for current goal achievement receive less support, although they may need it. The reason why important employees profit more when concealing could be that leaders are more likely to behave in terms of prevention to important employees as they fear the long-term negative consequences (e.g., long sick absence). Another possible explanation for our finding could be that leaders provide high staff care behavior to an important, irreplaceable employee as a positive leader–member relationship was established in the past when the employee contributed to the achievement of organizational goals. In turn, the leader feels obliged to return a favor and show higher staff care behavior when this employee shows early warning signals, although the employee is not disclosing. These explanations are in line with leader–member exchange and findings on reciprocity [52].

Fourth, contrary to our expectations, the negative effect of concealing did not decrease for leaders with higher health-oriented leadership skills. That is, although leaders with higher skills know that concealing is one typical reaction of employees in a conversation about noticed warning signals, they still show less staff care behavior toward non-disclosing than disclosing employees. To the best of our knowledge, this is the first study that investigated the effect of the interplay between skills and disclosure on staff care behavior. We may conclude that skills have a general overall effect independent of a specific situation (concealing vs. revealing).

This study makes important theoretical contributions. First, although awareness is considered an important precondition of behavior [8,9], awareness and behavior are distinct components of staff care. The transition from staff care awareness to behavior becomes especially important when employees’ health is at risk. After the perception of warning signals, leaders have to take appropriate health-related action. However, to the best of our knowledge, this is the first study that investigated potential obstacles in the transition between leaders’ awareness and subsequent behavior. By exploring antecedents, we add to the health-specific leadership literature [8], follow previous research calls [36], and advance the theory of healthy leadership as recommended by Rudolph et al. [35]. More precisely, we uncovered that employees’ disclosure, leaders’ skills, and the interplay between employees’ disclosure and leaders’ goal conflict play an important role in further staff care behavior. By adapting and applying the AMO framework [13,14], we offer a profound theoretical framework for future studies that want to investigate antecedents of leadership behavior. Second, we add new insights into the relationship between disclosure and leadership, as previous research has focused on leadership as a precondition for disclosing mental problems but has neglected the reverse causality (e.g., [6,39,53]). With an experimental study, we are able to demonstrate causal effects of employees’ disclosure on subsequent health-oriented leadership behaviors. By focusing on the effect of employee disclosure on leadership behavior, our work also complements a follower-centered perspective. In leadership research, the follower-centered perspective [24] emphasizes that employees actively influence leader behavior, especially when leaders rely on employee information.

### 5.1. Limitations and Future Research

Our findings should be interpreted against the background of our study’s limitations and avenues for future research. First, as our study used hypothetical vignettes and measured the intention of further staff care behavior, the external validity is limited, and it remains unclear how far our findings can be generalized to actual leadership behavior [54,55]. However, experimental studies are crucial in uncovering causal effects. In addition, laboratory and field studies often demonstrate consistent results [50], so we consider our experimental study as the first important starting point. In the next step, future studies may complement our findings in the field to strengthen external validity. A longitudinal design could replicate causal effects, capture staff care behavior after the perception of employees’ warning signals and starting the first conversation, and even uncover long-term effects of leaders’ skills, employees’ disclosure, and goal conflict on staff-care behavior. However, measuring the specific transition from awareness to behavior may be challenging. Conducting a retrospective study may resolve this difficulty but carries the risk of recall and hindsight bias [56]. Experimental designs minimize biases as variables can be controlled.

Second, our study consisted of written vignettes, and participants may have experienced a different level of affect depending on their imagination skills, potentially biasing our results. By carefully creating the vignettes and by checking the manipulation of disclosure and goal conflict in a pre-study, we tried to minimize this problem. Future studies may use role-plays instead of written materials so that, for example, the goal conflict can be experienced more intensively. Role plays may also help to better capture actual leadership behavior instead of an intention to behave. However, several other factors (e.g., outer appearance) may influence participants’ reactions when using role-plays.

Third, as the focus of our study was on the transition between staff care awareness and behavior in a specific situation, we do not know whether the studied antecedents apply to staff care in general. We may also have neglected other important situations where staff care is crucial to display in terms of prevention (e.g., designing working conditions independent of employees’ (mental) health status). However, we believe that the focus of our study is important, as many employees experience mental health issues at some point in their working life. The prevalence of mental disorders has even increased worldwide by 25% since the pandemic [57]. As leaders have to deal with employees’ mental health issues sooner or later, it is important to understand when leaders “further engage in staff care” in order to support affected employees in the best possible way. In addition, a better understanding of the transition between leaders’ perception of warning signals and their further behavior is also crucial, as staff care awareness represents an important precondition of staff care behavior [8,12].

Fourth, we did not include other factors that have been identified by past research as antecedents of staff care behavior (e.g., leaders’ strain, self care, crisis, organizational health climate, [9,10,12,36]). For example, it is conceivable that leaders with high skills show higher staff care behavior in an organization with a strong health climate as they have more opportunities for health-promoting actions that may facilitate the transfer from skills into action (e.g., a wide range of workplace health promotion offers, employee assistance programs, more support for designing healthy working conditions) so that future studies should incorporate this variable. However, we consider it a strength of our study that we focused on employees’ disclosure, leaders’ skills, and leaders’ experience of a goal conflict as they may especially be important after perceiving warning signals and starting a conversation with the respective employee.

### 5.2. Practical Implications

From a practical point of view, it is challenging for leaders to react appropriately to employees’ health-related warning signals because they do not know if their perception is correct and what behavior is appropriate. Our study offers important recommendations on how organizations can foster leaders’ further engagement in staff care behavior.

First, our results show that disclosure represents an important precondition for leaders that helps unfold their engagement in staff care behavior when they perceived employees’ warning signals. Therefore, organizations should aim at fostering employees’ openness to speak about mental health issues. This is also worthwhile as previous research shows that employees’ disclosure can enhance their well-being and work performance [39,40]. More precisely, organizations can try to reduce mental health-related stigma by providing knowledge about mental health, by protecting against discrimination, or by increasing the visibility of mental health issues in the organization, e.g., through voluntary outings of leaders and employees with recovered mental illness [58]. In addition, organizations can create a healthy climate by providing workplace accommodations, employee assistance, or return-to-work programs. Of course, all these interventions do not aim at naming specific illnesses but instead aim at creating a healthy climate with respect to privacy in which employees feel free to express emotional states, exhaustion, and the need for support. The practical implication for leaders is to be aware that concealing is a typical employee reaction and nevertheless keep on engaging in staff care behavior (e.g., keep on engaging in regular conversations, point out support possibilities). The practical implication for employees is to be aware of their responsibility to shape and influence leaders’ health-oriented behavior. If employees are able to put aside their fears of negative consequences and disclose emerging health issues to their leaders, they may be rewarded with additional support. This also underlines the shared responsibility for employees’ health as postulated in the HoL concept (see [8]).

Second, we found that participants in the intervention group showed higher staff care behavior than participants in the control group, so we may conclude that even a low-dose intervention that provides critical knowledge is suitable to foster staff care behavior. As many leaders feel uncomfortable or ‘out of their depth’ in conversations with employees about mental health [20,21,22], organizations should invest in enhancing health-oriented leadership skills. For example, as part of workplace health promotion, organizations can create short videos that provide knowledge to leaders about the most important health-related leadership issues. Although there is a growing body of evidence on the effectiveness of health-oriented leadership training [42,59,60,61], some programs are complex and time-consuming. Short-term, low-dose videos save costs and time and guarantee accessibility [62]. They can be used as a valuable addition to existing programs, as leaders’ schedules are often busy. We showed that even a small intervention had initial success in improving leaders’ skills, which in turn facilitated the transfer of perceiving employees’ warning signals (staff care awareness) to taking concrete health-promoting actions (staff care behavior). Staff care behavior, in turn, has been associated with better employee health, work performance, and satisfaction (e.g., [2,3]). Therefore, by investing in improving leadership skills, organizations help reduce the high burden arising from mental health issues.

## 6. Conclusions

When leaders perceive employees’ warning signals and worry that their employees’ health may be at risk (staff care awareness), leaders can take concrete health-promoting actions (staff care behavior) to prevent worsening or chronification. However, although awareness is considered an important precondition of behavior, awareness will not automatically lead to further actions. Until now, little was known about the factors facilitating or hindering the transfer from staff care awareness to concrete staff care behaviors. This is the first study that aimed at investigating antecedents of staff care behavior given the situation that a leader has perceived employees’ warning signals. Adapting the AMO framework, we investigated three antecedents: (1) leaders’ health-oriented leadership skills, (2) leaders’ motivation (depending on the experience of a goal conflict), and (c) employees’ disclosure as an opportunity and permission to take further actions. Our findings highlight that employees and leaders themselves influence the transfer from staff care awareness to further staff care behavior. Employees’ disclosure and leaders’ skills both had a positive effect on further staff care behavior. In addition, leaders’ experience of a goal conflict had no direct effect on staff care behavior but mitigated the negative effect of non-disclosure on staff care behavior. That is, leaders were more sensitive to indispensable employees, even when they did not open up. Our findings support the usefulness of applying the AMO framework [13,14] to studying antecedents of staff care behavior. More field research is needed to demonstrate if the findings can be generalized to actual leadership behavior and to understand what additional antecedents play a role in the transition from staff care awareness to behavior (e.g., organizational health climate). Organizations should aim at facilitating employees’ disclosure (e.g., by initiating antistigma campaigns) and improving leaders’ skills (e.g., knowledge about warning signals) to foster staff care behavior.

## Figures and Tables

**Table 1:** Sample characteristics.

		*n* (%)
Sex	Male	51 (56)
	Female	40 (44)
Age in Groups	<20	1 (1.1)
	20 to <30	77 (84.6)
	30 to <40	6 (6.6)
	40 to <50	0 (0)
	50 to 60	6 (6.6)
	≥60	1 (1.1)
Education	Secondary school	4 (4.4)
	Advanced technical college	1 (1.1)
	German higher education entrance qualification	48 (52.7)
	Polytechnic degree	1 (1.1)
	University degree	37 (40.7)

Note. *N* = 91.

**Table 2:** Means and Standard Deviations for Key Variables by Condition.

Skills:	Intervention Group (*n* = 47)				Control Group (*n* = 44)			
Disclosure	Concealing		Revealing		Concealing		Revealing	
Goal conflict:	No goal conflict	Goal conflict	No goal conflict	Goal conflict	No goal conflict	Goal conflict	No goal conflict	Goal conflict
	*M* (*SD*)	*M* (*SD*)	*M* (*SD*)	*M* (*SD*)	*M* (*SD*)	*M* (*SD*)	*M* (*SD*)	*M* (*SD*)
Staff care behavior	4.11 (0.61)	4.18 (0.67)	4.51 (0.43)	4.47 (0.50)	3.81 (0.69)	3.99 (0.61)	4.34 (0.66)	4.29 (0.49)

Note. *N* = 91.

**Table 3:** Three-Way Mixed RM-ANOVA for Disclosure, Skills, and Goal Conflict on Staff Care Behavior.

Predictor	d*f_Num_*	d*f_Den_*	*F*	*p*	η_p_^2^
Disclosure	1	89	91.15	0.000	0.501
Skills	1	89	4.01	0.048	0.043
Goal conflict	1	89	1.08	0.302	0.012
Skills × disclosure	1	89	0.72	0.398	0.008
Goal conflict × disclosure	1	89	6.90	0.010	0.072

Note. *N* = 91. d*f_Num_* indicates degrees of freedom numerator. d*f_Den_* indicates degrees of freedom denominator. η_p_^2^ indicates partial eta squared.

## Data Availability

The data are available from the authors upon reasonable request.
